# *Toxoplasma gondii* prevalence in food animals and rodents in different regions of China: isolation, genotyping and mouse pathogenicity

**DOI:** 10.1186/1756-3305-6-273

**Published:** 2013-09-21

**Authors:** Lin Wang, Hua-Wei Cheng, Kai-Quan Huang, Yuan-Hong Xu, Yong-Nian Li, Jian Du, Li Yu, Qing-Li Luo, Wei Wei, Ling Jiang, Ji-Long Shen

**Affiliations:** 1 Clinical Laboratory, the First Affiliated Hospital of Anhui University of Chinese Medicine, Hefei, Anhui Province 230031, People’s Republic of China; 2 Department of Parasitology, Provincial Laboratory of Microbiology & Parasitology and the Key Laboratory of Zoonoses Anhui, Anhui Medical University, Hefei, Anhui Province 230032, People’s Republic of China; 3 Department of Pharmacy, the Affiliated Provincial Hospital of Anhui Medical University, Hefei, Anhui Province 230001, People’s Republic of China; 4 Institute of Clinical Pharmacology, Anhui Medical University, Hefei, Anhui Province 230032, People’s Republic of China; 5 Clinical Laboratory, the First Affiliated Hospital of Anhui Medical University, Hefei, Anhui Province 230022, People’s Republic of China; 6 Department of Clinical Laboratory, Guiyang Medical College, Guiyang, Guizhou Province 550001, People’s Republic of China

**Keywords:** *Toxoplasma gondii*, Genotyping, Virulence, PCR-RFLP, Type China 1

## Abstract

**Background:**

Recent studies of *Toxoplasma gondii* isolates from animals in different regions of China have shown a limited genetic diversity and type China 1 was the dominant genotype of *T. gondii* prevalent in Chinese animals. However, little has been known concerning the isolation and genotyping of *T. gondii* circulating in chickens, pigs and rodents in China. The aim of the study was to characterize samples of *T. gondii* isolates obtained from naturally infected cats, pigs and free-range chickens slaughtered for human consumption in China.

**Methods:**

In the present study, brain tissues of 77 animals collected from different areas of China, including 24 free-range chickens (*Gallus domesticus*) , 13 voles (*Rattus flavipectus*), 23 pigs and 17 cats, were bioassayed in mice and viable *T. gondii* were isolated from the brains of eleven. These eleven *T. gondii* isolates were maintained in Kunming (KM) outbred mice and DNA isolated from tissues of infected mice was characterized using 11 PCR-restriction fragment length polymorphism (PCR-RFLP) markers: SAG1, SAG2, SAG3, BTUB, GRA6, c22-8, c29-2, L358, PK1, Apico, and CS3. Moreover, to determine mouse virulence of China 1 lineage of parasites, a TgCtgy5 genotype isolate was selected randomly and assessed in KM mice with different inoculation doses.

**Results:**

Results of genotyping revealed that ten isolates were type China 1 (ToxoDB PCR-RFLP genotype #9), and TgCksz1 was a new genotype that was reported for the first time designated here as ToxoDB PCR-RFLP #225. No clonal types I, II and III lineages were found. DNA sequencing of four introns (EF1, HP2, UPRT1 and UPRT7) and two genes (GRA6 and GRA7) from representative isolates confirmed the results of PCR-RFLP genotyping. The TgCtgy5 isolate was highly virulent in KM mice; all infected mice died of acute toxoplasmosis, irrespective of the inoculation dose. The results indicate that mouse virulent isolates of *T. gondii* are predominantly circulating in cats in China.

**Conclusions:**

*T. gondii* isolated from chickens, pigs, cats and rodents in different locations in China were genotyped and the results reconfirmed the limited diversity of *T. gondii* in China and showed that type China 1 lineage was dominant in this country.

## Background

*Toxoplasma gondii* is an important water- and food-borne obligate intracellular parasite, which infects almost all homoeothermic animals including humans, birds and domestic animals [1]. It has been estimated that up to 30% of the human population worldwide and 8% of population in China is chronically infected [2-4]. Felids are considered as the key in the transmission of *T. gondii* to humans and other animals because they are the only definitive hosts that excrete the environmentally resistant oocysts through defecation. Humans become infected postnatally by ingesting tissue cysts from raw or undercooked infected meat, or by consuming food or drink contaminated with *T. gondii* oocysts. However, only a small percentage of exposed adult humans or animals develop clinical disease. It is not clear why some hosts become ill with toxoplasmosis whereas most remain asymptomatic. Recently, attention has been focused on the genetic diversity among *T. gondii* isolates from apparently healthy and sick hosts. Severe cases of toxoplasmosis that have been reported in immunocompetent individuals are considered to be due to infection with atypical *T. gondii* isolates [5-7]. Type II strains are the most prevalent in humans and animals in Europe [8].

Globally, genetic diversity of *T. gondii* consists of six major clades originating from a small number of distinct ancestral lineages [8]. The clonal population structure with three major types I, II and III is more frequently observed in North America and Europe [9]. These three genotypes differ in their virulence and/or pathogenicity to mice. Type I strains are highly virulent, whereas type II and III strains are intermediately or non virulent. Moreover, infections with the atypical types A and X were also observed in wildlife in North America and have recently been described as members of a fourth clonal lineage, designated Haplogroup 12 [10]. However, subsequent studies using multilocus markers have revealed a greater genetic diversity of *T. gondii*, particularly isolates from humans and animals in South America [11,12]. These isolates have been historically considered as atypical in order to differentiate them from the dominantly described archetypes. Recent studies of *T. gondii* in humans and animals in Africa suggested the dominance of type II, III, Africa 1 and Africa 3 [13,14]. Some genotypes of *T. gondii* isolates from humans and cats in China have been previously reported, and the type China 1 (also known as ToxoDB#9) is widespread and likely the major lineage in mainland China [15-18].

However, the data of *T. gondii* isolates from food animals, such as chickens and pigs in China are limited. Food animals are the main meat source for human consumption in China, and risk factor analysis indicated that 30 to 63% of human infections can be attributed to the consumption of undercooked meat [19]. Serological and parasitological surveys in China indicate a high prevalence of infection of these animals [20,21], and a viable *T. gondii* isolate was isolated from tissues of pigs [22]. *T. gondii* infection in food animals continues to be a significant food safety problem, and has become to a major concern for Chinese consumers over the last decade. Additionally, toxoplasmosis also causes considerable economical loss to the animal husbandry industry worldwide due to increased mortality, abortion, and medical costs [23].

In the present study, we report the isolation and genotyping of *T. gondii* isolates from naturally infected cats, pigs, voles and free-range chickens in central and southwestern China using 11 polymerase chain reaction-restriction fragment length polymorphism (PCR-RFLP) markers. Additionally, the virulence of the isolates was determined in a mouse model. These results provide a new insight on the distribution of *T. gondii* genotypes and on *T. gondii* diversity in this country.

## Methods

### Ethics statement

All animals were treated in strict accordance to the guidelines for the Laboratory Animal Use and Care from Chinese CDC and the Rules for Medical laboratory Animals (1998) from Ministry of Health, China. The protocols were approved by the Institutional Review Board (IRB) of the Institute of Biomedicine at Anhui Medical University. All efforts were made to minimize animal suffering during the course of these studies.

### Animal samples and bioassay in mice

Experimental samples (brain tissues) were obtained from 24 free-range chickens (from Anhui province), 13 voles (from Hubei province), 23 pigs and 17 cats (from Guizhou province). To avoid any chance of cross-contamination, each sample was first separated from the body and placed into a labeled sterile plastic bag, and then the bag was placed in a cooler with ice packs to maintain the temperature at 4°C and taken to the laboratory. Samples were transported by train within 24 h of collection to the laboratory for *T. gondii* examination and isolation. All animals were anesthetized before being sacrificed.

The isolates were obtained by means of bioassays in 5-week-old Kunming (KM) female mice (specific pathogen free, SPF) following the previously described protocol [16,24]. Tissue homogenate was inoculated intraperitoneally into each of 5 mice. The mice were then monitored daily for illness. Peritoneal exudates were examined from mice for viable *T. gondii* isolates as soon as obvious clinical manifestations were observed. Survivors were killed on day 45 postinoculation and brains of all mice were microscopically examined for tissue cysts as a squash preparation as described [25]. The inoculated mice were considered infected with *T. gondii* when stages of the parasite (tachyzoites and/or tissue cysts) were demonstrated in their tissues. *T. gondii* isolates were cryopreserved in liquid nitrogen for future studies using homogenates of brains from infected mice, or viable tachyzoites from intraperitoneal fluids.

### DNA extraction and genotyping

DNA was extracted from ascitic fluids or tissues of all infected mice using QIAamp DNA Mini kit (Qiagen, Hilden, Germany) according to the manufacturer’s instructions. The purified DNA was dissolved in 20 μl of double-distilled water and stored at −20°C for multilocus genotyping studies. A negative control for DNA extraction was included for each group of five samples.

Genotyping of *T. gondii* isolates was performed using multilocus PCR-RFLP with 11 genetic markers as previously described: SAG1, SAG2, SAG3, BTUB, GRA6, c22-8, c29-2, L358, PK1, Apico [26,27] and marker CS3 [28]. Reference strains of *T. gondii* were also used in genotyping, including type I (GT1), type II (PTG), type III (CTG) and other strains (TgCgCa1, MAS, TgCatBr5, TgCatBr64, TgRsCr1). Briefly, the target DNA sequences were first amplified using a set of mixed external primers in a single reaction. Then multiplex products were 1:1 diluted in water and served as template DNA for nested PCR with internal primers for each marker separately. A known purified genomic DNA of *T. gondii* isolate (RH) was used as a positive control and DNA from non-infected mice were included as negative controls. To confirm successful amplification, 5 μl of the nested PCR products was run on a 1% agarose gel containing ethidium bromide prior to purification and digestion. The remaining products were purified using AxyPrep^TM^ PCR Cleanup Kit (Axygen, Union, USA) and digested with appropriate restriction endonucleases [29]. The restriction fragments were run by electrophoresis through a 2.5% ethidium bromide stained agarose gel for all markers except Apico, which required a 3% gel, and then visualized under UV light. A low DNA (25–700 bp) ladder (Ferments, Vilnius, Lithuania) was employed as size standard. The data were analyzed using ToxoDB (http://www.toxodb.org) database and compared with the reference strain profiles [30].

### SAG3 marker sequencing and phylogenetic analysis of *T. gondii* isolates

The nested PCR products for the marker SAG3 from *T. gondii* isolate TgCksz1 were sequenced in both directions using the internal *T. gondii* primers on an automated sequencer (Applied Biosystems, USA), as described previously [31]. Nucleotide sequences were analyzed and aligned using BioEdit Sequence Align-ment Editor [16]. For additional comparison, sequences from *T. gondii* RH strain (GenBank: JX218225), *T. gondii* PTG strain (GenBank: JX218226), and *T. gondii* CTG strain (GenBank: JX218227) available in the GenBank database (http://blast.ncbi.nlm.nih.gov/Blast.cgi) were also inputted.

To further characterize these isolates, three isolates (TgCtgy2, TgCtgy5 and TgPggy1) were selected randomly and sequences were generated at four introns (EF1, HP2, UPRT1 and UPRT7) and two dense granule proteins (GRA6 and GRA7) as described previously method [10]. The evolutionary history was inferred using a reticulated network by SplitsTree4 [32], combining the RFLP genotype results from this paper and predominant genotypes described to date in South America, Europe and North America.

### *T. gondii* isolate virulence in mice

To determine the pathogenicity of *T. gondii* isolates with type China 1 widespread in China, TgCtgy5 was selected randomly to study its virulence phenotype in mice. For this, tachyzoites were collected from peritoneal cavities of infected mice and purified as described [16]. Subsequently, parasites were diluted in phosphate buffered saline (PBS) and groups of ten five-week-old KM female mice were inoculated intraperitoneally with 10^1^, 10^2^, 10^3^, and 10^4^ tachyzoites, in a final volume of 200 μl PBS (per injection), as defined previously [33]. Five animals inoculated intraperitoneally with PBS were maintained as negative controls. The amount of tachyzoites was determined in a haemocytometer counting chamber. Mice mortality and morbidity was recorded daily for 45 d after infection, and survivors were killed under anesthesia and brains were examined for tissue cysts described as above. Meanwhile, the rest of the brains were tested for 529 bp repetitive fragment of *T. gondii* DNA by PCR using the primer pairs TOX4/TOX5 [34].

## Results

### Multilocus PCR-RFLP genotyping of *T. gondii* isolates

**Figure 1:**
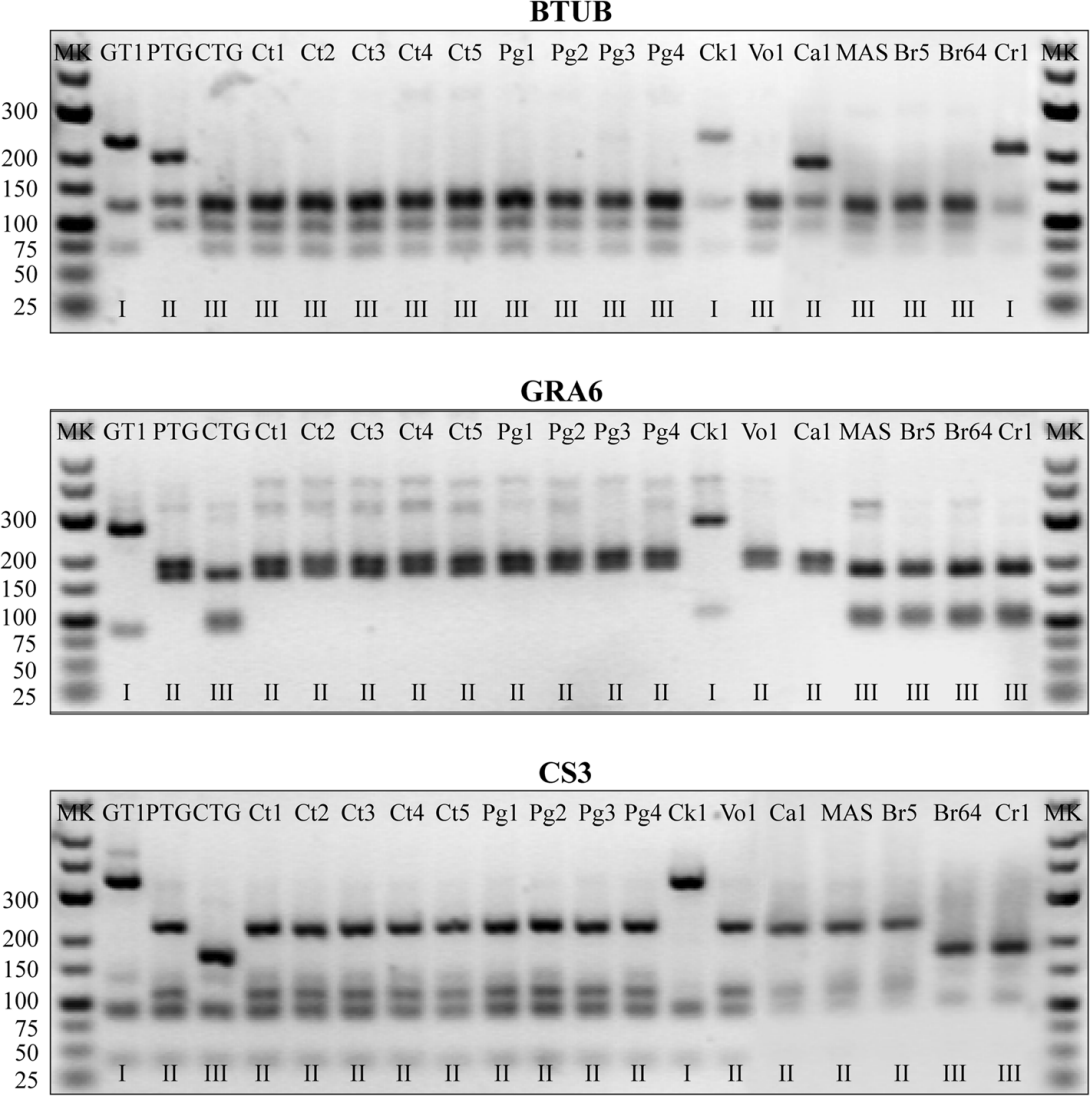
**Representative gel image of RFLP genotyping (markers BTUB, GRA6 and CS3).** Samples IDs are at the top of the gel images, genotype results are at the bottom. GT1, PTG, CTG, Ca1 (TgCgCa1), MAS, Br5 (TgCatBr5) and Br64 (TgCatBr64) are reference strains. Ct1: TgCtgy1; Ct2: TgCtgy2; Ct3: TgCtgy3; Ct4: TgCtgy4; Ct5: TgCtgy5; Pg1: TgPggy1; Pg2: TgPggy2; Pg3: TgPggy3; Pg4: TgPggy4; Ck1: TgCksz1; Vo1: TgVowh1. MK: molecular markers.

For the present study, 11 isolates of *T. gondii* isolated from 3 locations were genotyped. Five isolates were obtained from cats, four from pigs, one from chickens and one from voles by following essentially the procedures described previously [24]. To identify the genotype of the *T. gondii* isolates and improve the sensitivity of detection, a Mn-PCR-RFLP was employed and genotyping results for the 11 *T. gondii* isolates for all markers are shown in Figure 1 and Table 1. Two PCR-RFLP genotypes were detected and none of the isolates displayed a clonal genotype of types I, II or III.

**Table 1:** **Multilocus genotyping of *Toxoplasma gondii *isolates from different areas and hosts from China**

**Isolates**	**Host**	**Origin**	**SAG1**	**SAG2**	**SAG3**	**BTUB**	**GRA6**	**c22-8**	**c29-2**	**L358**	**PK1**	**Apico**	**CS3**	**ToxoDB PCR-RFLP #**	**Genotype**
GT1, reference	Goat	USA	I	I	I	I	I	I	I	I	I	I	I	**#10**	Type I
PTG, reference	Sheep	USA	II/III	II	II	II	II	II	II	II	II	II	II	**#1**	Type II
CTG, reference	Cat	USA	II/III	III	III	III	III	III	III	III	III	III	III	**#2**	Type III
TgCgCa1, reference	Cougar	Canada	I	II	III	II	II	II	μ-1^a^	I	μ-2 ^a^	I	II	**#66**	Atypical
MAS, reference	Human	France	μ-1 ^a^	II	III	III	III	μ-1 ^a^	I	I	III	I	II	**#17**	Atypical
TgCatBr5, reference	Cat	Brazil	I	III	III	III	III	I	I	I	μ-1 ^a^	I	II	**#19**	Atypical
TgCatBr64, reference	Cat	Brazil	I	μ-1 ^a^	III	III	III	μ-1 ^a^	I	III	III	I	III	**#111**	Atypical
TgRsCr1, reference	Toucan	Costa Rica	μ-1 ^a^	II	III	I	III	μ-2 ^a^	I	I	III	I	III	**#52**	Atypical
TgCtgy1,2,3,4,5	Cat	Guiyang, Guizhou	μ-1 ^a^	II	III	III	II	II	III	II	II	I	II	**#9**	China 1
TgPggy1,2,3,4	Pig	Guiyang, Guizhou	μ-1 ^a^	II	III	III	II	II	III	II	II	I	II	**#9**	China 1
TgCksz1	Chicken	Suzhou, Anhui	I	I	III	I	I	I	I	I	I	I	I	**#225**	
TgVowh1	Vole	Wuhan, Hubei	μ-1 ^a^	II	III	III	II	II	III	II	II	I	II	**#9**	China 1

^a^ u-1 and u-2 are new alleles that are different from the clonal type I, II and III alleles.

The present results showed that 10 isolates distributed in two provinces were grouped into one genotype (type China 1) based on the 11 markers analyzed. The genotype presented type II patterns at SAG2, GRA6, L358, PK1, c22-8 and CS3, but c29-2, SAG3, BTUB loci displayed a type III pattern and type I at the Apico locus. This genotype has already been found in three other hosts (cats, pigs and humans) but in voles for the first time from China. Type China 1 has been described not only in isolates from eastern China (Shandong, Jiangsu, Anhui and Zhejiang) but also in isolates from southern China (Guangdong), Central China (Henan and Hubei), northwest (Gansu) and southwest (Yunnan) of China. Therefore, the genotype is most likely a common genotype circulating in mainland China.

**Figure 2:**
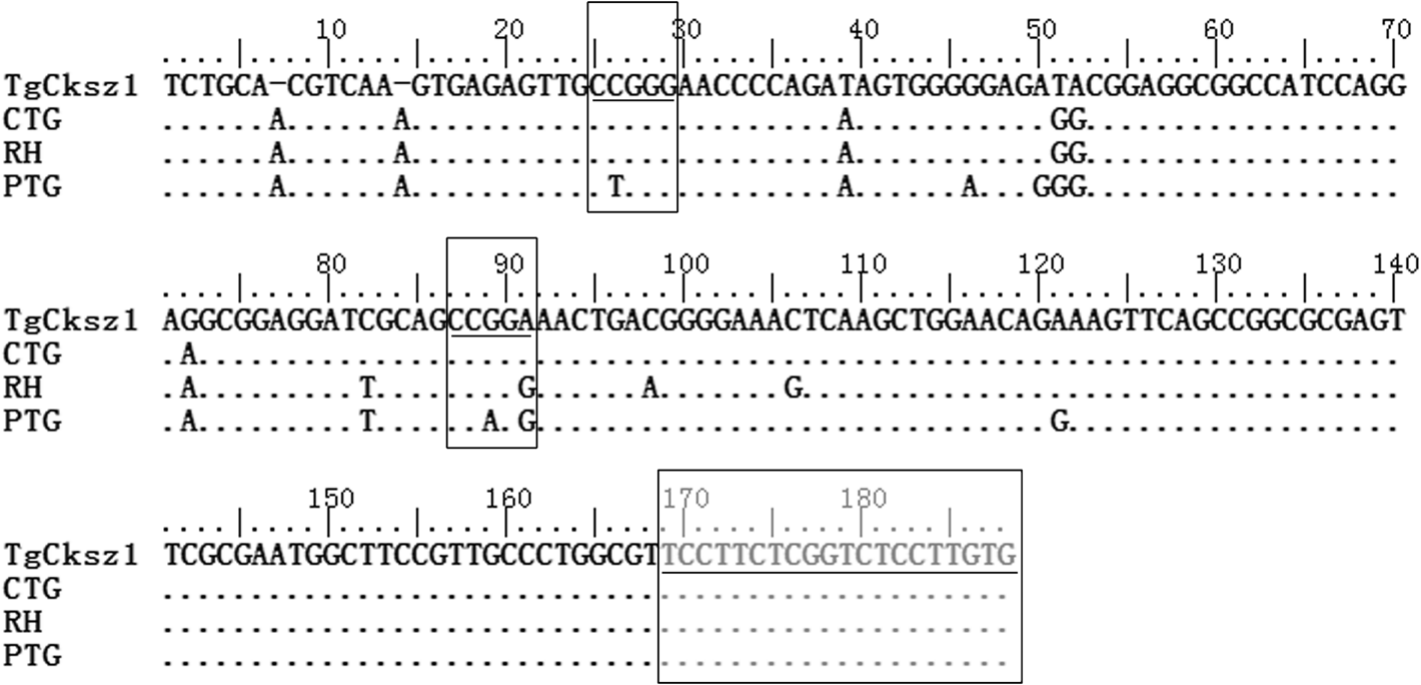
**Alignment of sequences of SAG3 fragment from *T. gondii *types I, II, and III and genotype #225.** Shaded box at the end of the sequences corresponds to hybridization sites of primer. Unshaded boxes correspond to the restriction sites for the enzyme NciI.

The remaining isolate (TgCksz1) was a variant of the archetypal type I, as all alleles observed were of type I except for the SAG3 locus where an allele of type III was observed. The SAG3 fragment from GT1 strain (type I) contained two restriction sites for the enzyme NciI, whereas the homologous PCR products from the CTG strain (type III) consisted of one restriction site. The nested PCR product for the marker SAG3 from PTG (type II) was not cleaved by NciI. The RFLP analysis of the SAG3 fragment from TgCksz1 revealed an identical pattern with the type III lineages (Table 1). The data were designated here as genotype #225. Furthermore, we determined the genotype by sequencing of a SAG3 gene of *T. gondii* TgCksz1, reconfirmed that the isolate contained a type III sequence without evidence of any additional mutations (Figure 2). The genotype was new and reported for the first time. This is the first report of genetic typing of *T. gondii* isolates from chickens and voles in China.

**Figure 3:**
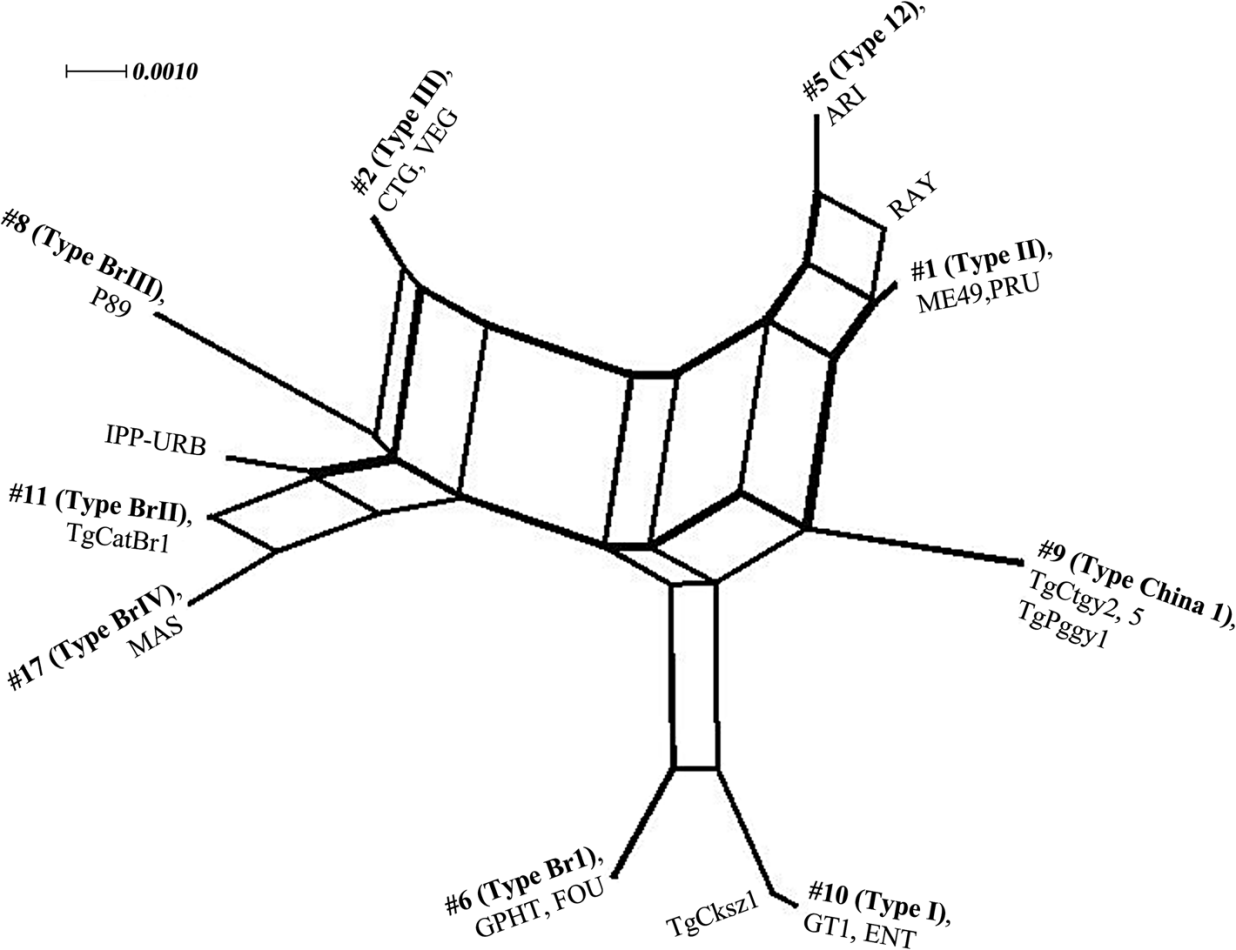
**Phylogenetic network analysis with representatives of *T. gondii *genotypes available in ToxoDB database.** Genotype number (#) and the representative strains are listed for each taxonomic branch.

The phylogenetic network of the representative isolates of *T. gondii* obtained from chickens, pigs and cats, together with previously published data were compared using SplitsTree4 software [32], with taxa positioned in the typical star-like network (Figure 3). A cluster analysis of the data showed that isolates TgCtgy2, 5 and TgPggy1 formed a single group, and a few major clonal lineages of *T. gondii* dominant in different geographical regions.

### Virulence of *T. gondii* isolates with type China 1

**Figure 4:**
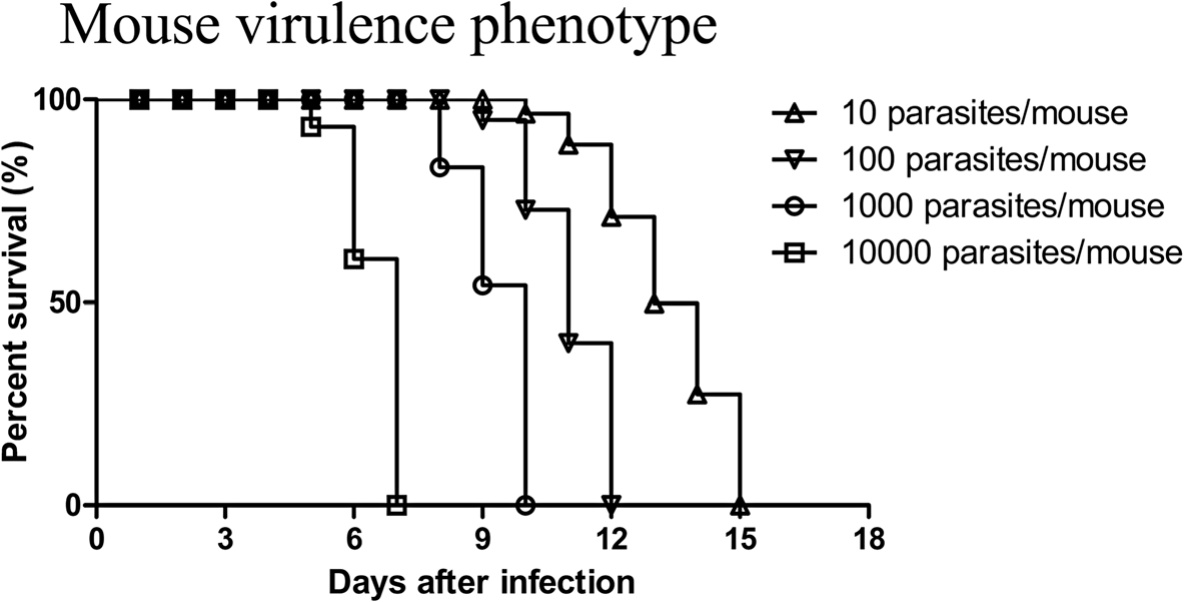
**Mouse survival rates with *T. gondii *genotype China 1.** Virulence of *T. gondii* type China 1 isolate TgCtgy5 was monitored in mice. Survival of KM mice was monitored for 45 d following intraperitoneal inoculation with different doses of tachyzoites indicated. Ten parasites of TgCtgy5 isolate killed 100% mice within 15 d.

The *in vivo* virulence of the *T. gondii* isolate TgCtgy5 in KM mice was determined using different tachyzoite doses. Mortality data after inoculation with tachyzoites are shown in Figure 4. All mice inoculated with tachyzoites died of toxoplasmosis between 5 d and 15 d post-infection, irrespective of the inoculation dose, indicating that the isolate was virulent in mice. The tachyzoites were found in peritoneal fluid of all dead mice, and the day of death was correlated with the inoculum dose. This result indicates that highly virulent *T. gondii* isolates are circulating in cats and they may cause more severe toxoplasmosis when spreading into human population.

## Discussion

We investigated the genetic diversity of *T. gondii* isolates from chickens, pigs, cats and voles from central and southwestern China. Among food animals, chickens and pigs are considered the most important reservoirs for *T. gondii* transmission and the isolation of *T. gondii* from chickens and pigs is a public health concern as human consumption of these meats continues to increase. The detection and identification of *T. gondii* from chickens, pigs and rodents facilitate our understanding of the epidemiology, population structure and virulence of *T. gondii*. Worldwide seroprevalence of *T. gondii* infection in chickens and pigs are summarized by Dubey [35,36], depending on the source of chickens and pigs and the sensitivity of diagnostic tests. In China, the seroprevalence of *T. gondii* infection in free-range chickens from 13 provinces/municipalities was 6.7%-47.3% [20], and that of pigs in most studies ranged between 12.0% and 53.4% [19,21,37], no genotyping data have been presented so far. These survey results indicated that infection with *T. gondii* in chickens and pigs is widespread in China, and that chicken and pork may be an important source for human infection with *T. gondii*.

Genetic diversity of *T. gondii* isolates has been an interesting and important research topic. In the present study, we isolated and completely genotyped 11 *T. gondii* isolates from chickens, pigs, cats and voles in eastern, southwest and central China, respectively. Analysis of the multilocus PCR-RFLP revealed 2 genotypes, one was identified in ten isolates (type China 1), while the other is represented by only one isolate and was described for the first time in the literature (Table 1). Our results indicated a limited diversity of *T. gondii* isolates from the animal population in China, a similar result as reported by other authors in studies of parasites isolated from different regions and host species [16,17,38,39]. All previous RFLP genotyping data and the present experiments of *T. gondii* isolates (totally 119) from China are summarized in Table 2. The compiled results indicated that type II and type III are rare in China. However, the type China 1 and type I accounted for 73.9 and 10.9%, respectively. The genotype China 1 has already been described in several hosts such as pigs, cats and humans, but this is the first report of this genotype in voles. Among intermediate hosts, infected rodents are considered the most important sources of *T. gondii* infection for cats. The type China 1 lineage was found for vole isolates in the present study, reconfirming that this lineage is the most common and widespread in different hosts and geographical locations in China. Overall, our current study is in agreement with previous report that *T. gondii* type China 1 is the dominating genotype in animals as well as humans within China [16]. The limited data on *T. gondii* isolates detected in food animals and wildlife, like pigs or voles, seem to reflect the dominant *T. gondii* type China 1 which is commonly found in livestock, poultry and humans. Interestingly, this genotype has also been identified in *T. gondii* isolates of dogs from Sri Lanka and Colombia [40,41], chickens from Brazil [27] and of sheep from the United States [42], suggesting that such a genotype might be widespread from Asia to North and South America. Further analysis of these *T. gondii* isolates at the DNA sequence level may help us understand the origin and migration of this parasite among different continents.

**Table 2:** **Genetic PCR-RFLP types of *T. gondii *in animals and humans in China**

**Host**	**No. of isolates**	**ToxoDB PCR-RFLP genotypes**										**References**
		**#10 (Type I)**	**#1 (Type II)**	**#2 (Type III)**	**#3 (Type II-variant)**	**#9 (China 1)**	**#18**	**#204**	**#205**	**#213**	**#225**	
Cats	72	1	0	0	0	65	2	0	4	0	0	[15,16,38] and this study
Pigs	33	12	0	0	0	19	0	0	0	2	0	[22,39,43] and this study
Sheep	1	0	0	0	1	0	0	0	0	0	0	[17]
Rabbits	1	0	0	1	0	0	0	0	0	0	0	[44]
Wild birds	3	1	0	0	2	0	0	0	0	0	0	[45]
Chickens	1	0	0	0	0	0	0	0	0	0	1	This study
Voles	1	0	0	0	0	1	0	0	0	0	0	This study
Humans	7	2	1	0	0	3	0	1	0	0	0	[16,17]
Total	119	13 (10.9 %)	1 (0.8 %)	1 (0.8 %)	3 (2.5%)	88 (73.9 %)	2 (1.7 %)	1 (0.8 %)	4 (3.4 %)	2 (1.7 %)	1 (0.8 %)	

There is scarce information concerning the isolation and genotyping of *T. gondii* isolates from chickens in China. Because free-range chickens become infected mainly by feeding from ground/soil contaminated with oocysts, the prevalence of *T. gondii* in this host has been widely used as an indicator of the strains prevalent in the environment. In addition, tissues of infected chickens are considered a good source of infection for cats, humans and other animals. In the present investigation, the isolate TgCksz1 (genotype ToxoDB#225) isolated from a chicken in Suzhou city is similar to the genotype of isolate TgCkBr136 (genotype ToxoDB#41), which was collected from chickens in the state of Rondönia in Brazil and identified by Dubey [46]. The genotype ToxoDB#225 was a variant of the type I with a type III genotype at the SAG3 loci. Meanwhile, the genotype ToxoDB#41 showed type II pattern at GRA6 and the remaining 9 loci types were identical to type #225, suggesting that they are phylogenetically related. In addition, the genotype #41 was also identified from newborns in the Minas Gerais state in Brazil [47]. These results suggest that whether this isolate TgCksz1 has been imported into China or is endemic remains unknown based on the genetic data. However, as indicated above, genetically similar isolates were observed in distant regions like these should be investigated in future studies. To our knowledge this is the first report of genetic typing of *T. gondii* isolates from chickens from China. None of the isolates displayed a clonal genotype (type I, II and III).

Before the recognition of the three *T. gondii* genotypes, isolates were phenotypically classified as mouse virulent or avirulent. Phenotypically, type I strains are uniformly lethal to outbred mice and type II and III strains are significantly less virulent [11]. In this study, we observed variability in the mouse virulence of the *T. gondii* isolates with the genotype China 1, including virulent isolates and intermediately isolates. The TgCtgy5 isolate was lethal to outbred KM mice; all infected mice died of acute toxoplasmosis, regardless of the dose used. However, all mice inoculated with 1 × 10^3^ tachyzoites of the isolate TgCtgy2 (type China 1) died but two survived acute infection and cysts were found in the brain tissues of the survivals. The differences of biological features among the isolates sharing the common genotype must not be neglected and it may be possible that the genetic markers used in this study are incapable of reflecting possible phenotypic differences between the isolates under question [48]. Also, in the previous report, we showed that mouse virulence and genetic type are not strictly correlated because two *T. gondii* isolates (TgCtwh3 and TgCtwh6) share the common genotype but have markedly different mouse virulence, including CS3 locus (type II). Similar phenomenon could also be found in other studies on type China 1 isolates [38]. The marker CS3, located on chromosome VIIa of *T. gondii*, was previously reported to be linked with the acute virulence in mice of *T. gondii* and was also used to determine its association to parasite virulence in mice [49]. Several studies indicated that isolates with the alleles I and II at the CS3 locus are strongly associated with virulence in mice [28,31]. The Chinese isolate of *T. gondii* TgCtwh6, however, has a low virulence to mice but shares the allele II at the CS3 locus. These results suggest that better genetic markers are needed to correlate pathogenicity of *T. gondii* in animals and humans.

## Conclusions

The present study is the first report of *T. gondii* isolates collection and genotyping obtained from chickens and voles, enriching the limited *T. gondii* genotype database in China. The genetic structure here found in the isolates from chickens, pigs, cats and rodents corroborates the findings of previous studies that *T. gondii* has a limited diversity in China. In addition, the results provided preliminary data for further approaches of epidemiology of *T. gondii* in food animals and wildlife and analysis of population genetics of *T. gondii* organisms.

## Competing interests

The authors declare that they have no competing interests.

## Authors’ contributions

JLS, LJ, LW and HWC conceived and designed the study, and critically revised the manuscript. LW, HWC, KQH, YHX and YNL performed the experiments, analyzed the data and drafted the manuscript. JD, LY, QLL and WW participated in the analysis and interpretation of data. All authors have read and approved the final manuscript.
